# Effect of consolidation chemotherapy following definitive chemoradiotherapy in patients with esophageal squamous cell cancer

**DOI:** 10.1038/s41598-017-17254-9

**Published:** 2017-12-04

**Authors:** Sheng-Xi Wu, Xu-Yuan Li, Hong-Yao Xu, Qi-Ni Xu, He-San Luo, Ze-Sen Du, He-Cheng Huang, Zhi-Yong Wu

**Affiliations:** 1 Department of Radiotherapy, Shantou Central Hospital, Shantou, Guangdong, China; 2 Department of Medical Oncology, Shantou Central Hospital, Shantou, Guangdong, China; 3 Department of Respiratory Medical Oncology, Cancer Hospital of Shantou University Medical College, Shantou, Guangdong, China; 4 Department of Surgical Oncology, Shantou Central Hospital, Shantou, Guangdong, China; 5 Department of Diagnosis and Treatment Center of Breast Diseases, Department of Surgical Oncology, Shantou Central Hospital, Shantou Central Hospital, Shantou, Guangdong, China

## Abstract

Definitive chemoradiotherapy (dCRT) is a treatment option for patients with localized esophageal squamous cell carcinoma (ESCC). We investigated consolidation chemotherapy (CCT) in patients with ESCC who attained clinical complete response after dCRT. Between January 2009 and December 2012, medical records of ESCC patients treated with dCRT were retrospectively reviewed, and those who attained CCR were identified. Progression-free survival and overall survival rates were estimated by the Kaplan-Meier method. The Cox regression model was used to determine prognostic factors. Of the 522 patients treated with dCRT, 209 patients achieved CCR, with 67 receiving consolidation chemotherapy (the CCT group) and 142 receiving dCRT alone (the control group). CCT did not prolong progression-free survival (33.0 *vs* 18.0 months, *P* = 0.07, *HR* = 0.70, 95% *CI*, 0.48–1.04); however, CCT improved the median overall survival (53.4 *vs* 27.0 months, *P* = 0.04, *HR* = 0.67, 95% *CI*, 0.44–0.99) compared with dCRT alone. CCT remained a favorable prognostic factor for overall survival in a multivariate analysis (*HR* = 0.59, *P* = 0.02); however, a propensity score analysis failed to show an additional overall survival benefit with CCT. In the present analysis, CCT did not improve progression-free survival but may have extended overall survival in ESCC patients who achieved complete clinical response after dCRT.

## Introduction

Esophageal cancer is the fifth most common cause of cancer-related mortality in China^1^. Although the incidence of esophageal squamous cell carcinoma (ESCC) has decreased in western countries^2^, ESCC remains the main pathological subtype in China. A large prospective phase III trial recently established surgery combined with preoperative chemoradiotherapy as a standard care treatment for patients with resectable, locally advanced esophageal cancer^3,4^. However, this trimodal therapy has not been widely adopted in western countries^5^. In addition, only 23% (84/359) of patients included in the trial had squamous cell carcinoma and thus did not represent the routine clinical practice in some countries where ESCC remains prevalent.

Definitive chemoradiotherapy (dCRT) remains an option for potentially curable esophageal cancers, particularly for those with squamous cell carcinoma histology^6,7^. Although dCRT is not widely accepted, it is an optimal strategy for medically unfit patients or those who decline surgery. In patients with squamous cell histology, regular follow-up and salvage surgery may be a good strategy for those who responded well to initial dCRT^8^. Unfortunately, the survival outcome of curable esophageal cancer is poor, with a 5-year overall survival rate between 15% and 40%^9^.

Consolidation chemotherapy (CCT) after initial treatment is intended to improve cancer patient outcomes and has shown efficacy in some cancers, such as cervical^10^ and non-small-cell lung cancer^11^. However, reports on CCT for patients with esophageal cancer after dCRT are rare. Therefore, we retrospectively reviewed medical records of ESCC patients treated with dCRT, and separated them into the CCT group and the control group (without CCT after dCRT) to determine the role of CCT in ESCC patient survival.

## Results

Between January 2009 and December 2012, 522 ESCC patients who received dCRT were identified, and their medical records were reviewed. Of the 522 patients, 209 (40%) attained CCR. Sixty-seven patients received consolidation chemotherapy (the CCT group) and 142 received dCRT alone (the control group). The median ages at diagnosis for each group were 59 (range: 43–79) and 66 (range: 43–89) years, respectively. As shown in Table 1, patient characteristics between the two groups were well-balanced.

**Table 1:** Patient characteristics.

	CCT (N = 67)	%	Control (N = 142)	%	*P* value
**Age (median)**	59		66		
≤60 years	27	40.3	40	28.2	0.08
>60 years	40	59.7	102	71.8	
**Gender**					0.106
Male	53	79.1	97	68.3	
Female	14	20.9	45	31.7	
**Location**					0.705
Cervical	8	11.9	10	7.0	
Upper	13	19.4	30	21.1	
Middle	39	58.2	86	60.6	
Lower	7	10.4	16	11.3	
**Length (cm)**					0.136
≤5	22	32.8	62	43.7	
>5	45	67.2	80	56.3	
**Nodes**					0.185
Negative	17	25.4	49	34.5	
Positive	50	74.6	93	65.5	
**T**					0.357
1–2	22	32.8	56	39.4	
3–4	45	67.2	86	60.6	
**Stage**					0.26
I	11	16.4	30	21.1	
II	23	34.3	59	41.5	
III	33	49.3	53	37.3	

CCT, consolidation chemotherapy.

Following dCRT, 34 patients received two cycles of CCT consisting of the same 5-FU and cisplatin regimen applied in the dCRT course, and 33 received two cycles of docetaxel (60–70 mg/m^2^, day 1) with cisplatin (20–25 mg/m^2^, day 1–3) or nedaplatin (60–70 mg/m^2^, day 1). No deaths occurred during the treatment, and no patients resorted to salvage surgery.

**Figure 1:**
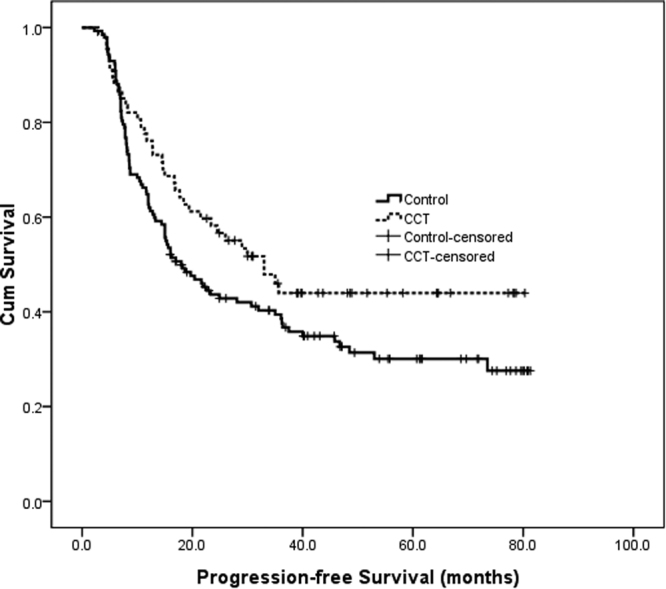
Progression-free survival curve of the CCT and chemoradiotherap group.

The median progression-free survival times were 33.0 months (95% CI, 12.4–23.6 months) and 18.0 months (95% CI, 22.2–43.7 months) in the CCT and control groups (*χ* ^2^ = 3.23, *P* = 0.07, *HR* = 0.70, 95% *CI*, 0.48–1.04, Fig. 1), respectively.

No differences in total progression events were observed between the two groups, with 94 (66.2%) of 142 occurring in the control group and 36 (53.7%) of 67 in the CCT group (*χ* ^2^ = 3.00, *P* = 0.083). A similar finding was observed for local recurrence events, with 32 of 142 (22.5%) occurring in the control group versus 21 (31.3%) of 67 in the CCT group (*χ* ^2^ = 1.86, *P* = 0.17). However, fewer patients had distant metastases in the CCT group than in the control group (15 [22.4%] of 67 versus 62 [43.7%] of 142, *χ* ^2^ = 8.85, *P* = 0.003).

**Figure 2:**
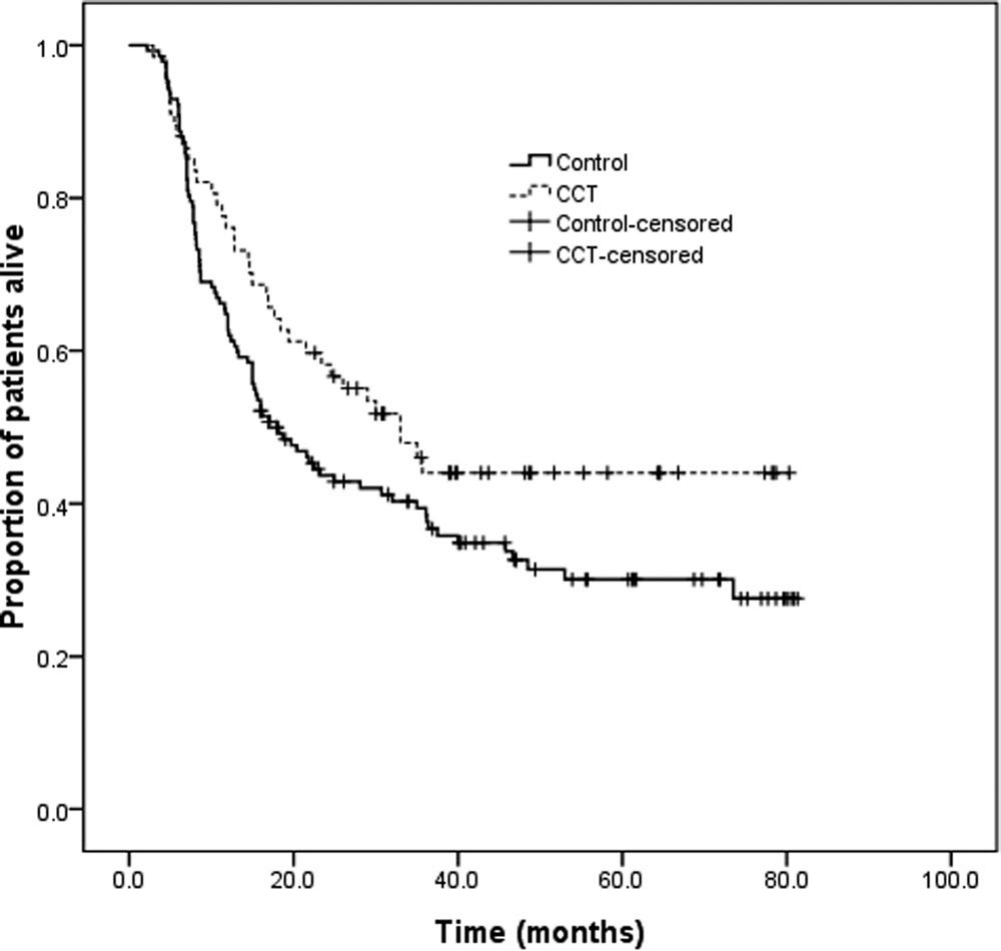
Overall survival curve of the CCT and chemoradiotherap group.

The median overall survival times were 53.4 months (95% CI, 25.4–81.3 months) and 27.0 months (95% CI, 17.3–36.7 months) for the CCT and control groups (*χ* ^2^ = 3.87, *P* = 0.04, *HR* = 0.67, 95% *CI*, 0.44–0.99, Fig. 2), respectively. The 1-, 2- and 3-year overall survival rates were 67.3%, 38.9%, and 30.2% in the CCT group and 62.8%, 34.4%, and 26.4% in the control group, respectively. Multivariate analysis showed that CCT (*HR* = 0.59, 95% *CI*, 0.40–0.98, *P* = 0.02) and disease stage (*HR* = 1.65, 95% *CI*, 1.06–2.25, *P* = 0.01) were prognostic factors for overall survival. In the univariate and multivariate analyses, the HRs for age were 1.03 (95% *CI*, 1.00–1.05, *P* = 0.02) and 1.52 (95% *CI*, 0.99–2.32, *P* = 0.05), respectively.

After matching (67 patients per treatment group), the median overall survival was 53.4 months (95% *CI*, 25.4–81.3) for the CCT group versus 30.1 months (95% *CI*, 9.0–51.2) for the control group (*P* = 0.23). The HR was 0.75 (95% *CI*, 0.47–1.20).

The most common grade 3 or greater adverse events to occur during CCT were neutropenia, nausea, and stomatitis in 17.9% (12 patients), 16.4% (11 patients), and 10.4% (7 patients), respectively.

## Discussion

Definitive chemoradiotherapy is primarily indicated for patients with resectable esophageal cancer who refused surgery or for patients with a locally advanced unresectable disease. Moreover, several clinical trials have shown comparable survival outcomes between dCRT and surgery^6,7,12^. In a small prospective randomized trial, dCRT obtained a better 5-year survival rate of 50% compared with 29.4% from surgery, although the difference was not statistically significant^12^. In practice, definitive chemoradiotherapy is recommended as a choice of care in patients with esophageal cancer who refused surgery^8,13^.

After definitive dCRT, patients who attained a complete response were followed closely, and salvage surgery was suggested when indicated^14^. Consolidation chemotherapy after dCRT has been tested with other tumor types in some large prospective clinical trials^15,16^, but most failed to show efficacy. To our knowledge, no prospective studies have reported the role of consolidation chemotherapy after dCRT in esophageal cancer.

In this retrospective study, consolidation chemotherapy was associated with a longer progression-free survival compared with dCRT alone. The absolute difference in progression-free survival was remarkable at 15 months, although it was not significant (*P* = 0.07). One reason might be that the small sample size of our study was underpowered to detect the difference.

In contrast, a significant prolonged 26.4-month overall survival was observed. The possible contributing factor that consolidation chemotherapy reduced distant metastases was also observed in other trials^17^; however, this result should be interpreted cautiously, because bias is inevitable in retrospective studies. Treatment for relapsed disease may also impact overall survival. Moreover, in the propensity score matching analysis, CCT yielded no survival advantage, despite the CCT group’s longer survival time. Thus, it appears that selection bias was an important confounder. However, the matching analysis included only a small number of patients. A prospective randomized trial will likely solve these problems.

Pathological complete response is considered a surrogate marker for favorable prognosis in esophageal cancer^18^, indicating a better response to therapy. However, for patients under non-operative management, validating pathological complete response was impossible. Fang *et al*. reviewed CT scans, PET/CT images and endoscopy results to define clinical complete response in patients with esophageal cancer post-chemoradiotherapy^19^. In contrast, complete clinical response in our study was evaluated by a CT scan and barium meal examination; however, under this assessment, patients who were deemed to have a complete clinical response had significantly better overall survival than those who had a partial response (median overall survival of 13 months). Therefore, this assessment may effectively test the role of consolidation chemotherapy in a treatment-sensitive population.

The chemotherapy delivered in our study could be improved. First, doubled 5-FU and cisplatin is the chemotherapy backbone of the chemoradiotherapy in our daily practice. However, tripled regimens have been investigated and show promising results. In a prospective phase 1/2 study, dCRT using docetaxel, nedaplatin, and 5-fluorouracil showed strong activity with an 82.1% complete response rate and a progression-free survival of over 40 months in patients with stage IB to IV^20^. Second, 5-FU or docetaxel plus cisplatin were administered as consolidation therapy in our study. Several retrospective studies have revealed other promising combinations. Liu *et al*. reported that a regimen of paclitaxel plus cisplatin showed better efficacy in progression-free survival with tolerable toxicities^21^.

The current study had several limitations. The retrospective design and short follow-up limited the strength of the results. In addition, assessing complete clinical response by CT scan and barium meal examination was not optimal.

In conclusion, the present study indicated that CCT reduced distant metastases but failed to improve progression-free survival. However, it did show potential to improve the overall survival of ESCC patients who achieved CCR after dCRT. These findings indicate the need to prospectively evaluate the benefit of CCT in this population.

## Patients and Methods

This study was approved by the Ethics Committee of the Shantou Central Hospital, Shantou, China. All experimental protocols were approved by the committee. All procedures were performed in compliance with the approved guidelines. Informed consent was obtained from all patients prior to treatment.

Between January 2009 and December 2012, patients with esophageal cancer who received chemoradiotherapy at the Department of Radiotherapy, Shantou Central Hospital, China, were screened for study enrollment. Inclusion criteria were as follows: pathologically proven ESCC, stage I to III disease, standard definitive treatment protocol, and tumor disappearance after initial dCRT. Exclusion criteria were as follows: adenocarcinoma or other pathological types, stage IV disease, palliative or adjuvant radiotherapy, and evidence of residual tumor after dCRT.

Definitive CRT was administered to patients with technically unresectable cancer or those who refused surgery. Each treatment field encompassed the tumor bed with 3-cm proximal and distal margins and 1-cm lateral margins. Patients received a total of at least 50.4 Gy irradiation. Daily treatment with 2-Gy external-beam irradiation was delivered by a 10-MV linear accelerator with parallel opposing fields using anterior and posterior portal arrangements, which were changed to an oblique portal arrangement after 40 Gy. All patients received 5-fluorouracil (5-FU) and cisplatin-based chemotherapy concurrently with irradiation. The concurrent chemotherapy regimen consisted of two cycles of 5-FU (750 mg/m^2^, days 1–4) and cisplatin (20–25 mg/m^2^, days 1–3) every 21 days.

All patients were evaluated 3–4 weeks after completing dCRT (and CCT) and every 3–6 months for the next 5 years. The first year of follow-up evaluation included a physical examination, blood test, barium meal examination, and CT scan of the neck, chest, and abdomen. The following regular evaluations included a physical examination, blood test, and barium-meal examination.

Information on patient age, gender, work-up, treatment, and follow-up was extracted from their medical records. Patient stages were determined according to the 6th TNM classification.

A progression-free event was defined as the first documented radiographic evidence of progressive disease, or death from any cause. Progression-free survival and overall survival rates were estimated by the Kaplan-Meier method. Differences between survival rates were assessed by the log-rank test. A chi-square test was used for categorical data. A *P* value < 0.05 indicated a significant difference. All *P* values were two-sided. The Cox regression model was used to identify prognostic factors.

The propensity score analysis (including age, sex, tumor length, tumor location, T stage, nodal status, and stage) was performed using the one-to-one nearest neighbor method. R software (version i386 3.3.2) was used for matching with the *MatchIt* package.
